# Using Oculomotor Features to Predict Changes in Optic Nerve Sheath Diameter and ImPACT Scores From Contact-Sport Athletes

**DOI:** 10.3389/fneur.2021.584684

**Published:** 2021-03-04

**Authors:** Hrishikesh M. Rao, Sophia Yuditskaya, James R. Williamson, Trina R. Vian, Joseph J. Lacirignola, Trey E. Shenk, Thomas M. Talavage, Kristin J. Heaton, Thomas F. Quatieri

**Affiliations:** ^1^Human Health & Performance Systems Group, Massachusetts Institute of Technology Lincoln Laboratory, Lexington, MA, United States; ^2^Counter-Weapons of Mass Destruction Systems Group, Massachusetts Institute of Technology Lincoln Laboratory, Lexington, MA, United States; ^3^Advanced Radio Frequency Techniques & Systems Group, Massachusetts Institute of Technology Lincoln Laboratory, Lexington, MA, United States; ^4^Department of Biomedical Engineering, Weldon School of Biomedical Engineering, Purdue University, West Lafayette, IN, United States; ^5^Department of Electrical and Computer Engineering, Purdue University, West Lafayette, IN, United States; ^6^Military Performance Division, U.S. Army Research Institute of Environmental Medicine, Natick, MA, United States

**Keywords:** eye movements, optic nerve sheath diameter, neurocognitive testing, contact sports, athletic season

## Abstract

There is mounting evidence linking the cumulative effects of repetitive head impacts to neuro-degenerative conditions. Robust clinical assessment tools to identify mild traumatic brain injuries are needed to assist with timely diagnosis for return-to-field decisions and appropriately guide rehabilitation. The focus of the present study is to investigate the potential for oculomotor features to complement existing diagnostic tools, such as measurements of Optic Nerve Sheath Diameter (ONSD) and Immediate Post-concussion Assessment and Cognitive Testing (ImPACT). Thirty-one high school American football and soccer athletes were tracked through the course of a sports season. Given the high risk of repetitive head impacts associated with both soccer and football, our hypotheses were that (1) ONSD and ImPACT scores would worsen through the season and (2) oculomotor features would effectively capture both neurophysiological changes reflected by ONSD and neuro-functional status assessed via ImPACT. Oculomotor features were used as input to Linear Mixed-Effects Regression models to predict ONSD and ImPACT scores as outcomes. Prediction accuracy was evaluated to identify explicit relationships between eye movements, ONSD, and ImPACT scores. Significant Pearson correlations were observed between predicted and actual outcomes for ONSD (Raw = 0.70; Normalized = 0.45) and for ImPACT (Raw = 0.86; Normalized = 0.71), demonstrating the capability of oculomotor features to capture neurological changes detected by both ONSD and ImPACT. The most predictive features were found to relate to motor control and visual-motor processing. In future work, oculomotor models, linking neural structures to oculomotor function, can be built to gain extended mechanistic insights into neurophysiological changes observed through seasons of participation in contact sports.

## 1. Introduction

Traumatic brain injury (TBI) remains an important public health concern in high school sports and the military. Rates of diagnosed concussion in the military are quite high, with more than 413,858 documented cases of TBI since 2000 (1). Nearly 1.2 million student athletes participate in football every year (2) and the number of sport-related concussions have increased from 300,000 (3) to 3.8 million (4) in the last two decades. Sub-concussive head impact exposures are particularly difficult to detect as they do not elicit immediately identifiable symptoms and therefore, athletes may continue to participate risking subsequent injury. Yet, there is growing evidence that repetitive head impact exposure leads to neurophysiological (e.g., neural activation) and neurological (e.g., inflammation) changes (5–8). The ability to objectively quantify the physiological and cognitive impact of both concussive and sub-concussive head impact has become increasingly important.

Long-term consequences are associated with repetitive head impact exposure. Most prominently reported is chronic traumatic encephalopathy (CTE), which is closely tied to participation in contact sports, such as American football and soccer, among others (9). Symptoms associated with the neuropathological findings from those documented with CTE can include difficulty concentrating, depression, and personality changes in the early stages, potentially leading to memory loss, Alzheimer's Disease, and dementia in the later stages (10, 11). There are still open questions about risk factors for CTE and further prospective research is needed to track the development of CTE based on quantified repetitive head impacts.

Needed are markers of head impact-induced neurophysiological changes that can reliably detect subtle, clinically-meaningful gradations of change from baseline, which can offer new options for assessment of brain injury. One such potential marker is intracranial pressure (ICP), which is most commonly measured and documented only in moderate-to-severe TBI. Inflammatory and neurotoxic processes that occur following brain trauma, as well as obstruction of CSF flow caused by the injury, are major contributors to TBI-induced increases in ICP (12). Although little is known about the specific etiology of ICP in mild TBI and repetitive sub-concussive head impact exposure, the expected physiological response under intact autoregulation (per the Monro-Kellie doctrine) suggests that even small increases in ICP from baseline may be diagnostically informative (13). Thus, both the absolute values and relative changes in ICP can potentially serve as a graded, as opposed to a binary, marker for a continuous spectrum of brain injury assessment.

As current methods for measuring ICP directly are highly invasive, efforts have been made to identify alternate approaches. One such measure uses ultrasound or brain computed tomography imaging to provide a measure of optic nerve sheath diameter (ONSD). Because the optic nerve is an extension of the dura mater with direct continuity between the intracranial subarachnoid space and that of the optic nerve, an increase in CSF pressure, induced by increased ICP, will also cause the optic nerve to increase in diameter (14, 15). Several studies have demonstrated this relationship between ultrasound-measured ONSD and increased ICP, specifically focusing on patients with TBI (16). With much empirical evidence demonstrating this mechanistic link, ONSD has proven to be a valuable clinical proxy for assessing ICP. However, this approach is limited by the need for an expert viewer to read the ultrasound measurements and visually interpret the ONSD value (17), which prevents more widespread use beyond clinical settings. Additionally, while ONSD provides sensitive detection of head impact-induced increases in ICP, a response that affects the brain globally, it does not provide a specific mechanistic understanding of the type of brain injury. Therefore, additional tools for detecting and understanding head impact-induced changes are needed to guide rehabilitation and return-to-play/duty decisions.

ImPACT (Immediate Post-concussion Assessment and Cognitive Testing), a computerized cognitive assessment battery, offers insight into a variety of neurocognitive functions for TBI diagnosis and phenotyping (18, 19). In studies involving high school and college athletes, the ImPACT showed high sensitivity and specificity at classifying individuals who had a concussion (20). Further, ImPACT has been shown to detect sub-concussive impact exposures through a sports season (21). Despite its versatility, ImPACT has several drawbacks with regards to assessment for return-to-play or injury rehabilitation. As with almost all cognitive assessment tools, the ImPACT is susceptible to an individual's level of motivation to perform at his or her best on any given assessment (22–24). Additionally, the testing paradigm does not provide mechanistic insights into the anatomical site of injury, the impacted neural pathways, or the potential relationship between test performance and ICP. Therefore, use of the ImPACT to guide treatment and rehabilitation efforts remains a challenge. A methodology that provides objective, diagnostic information to guide intervention would be a complement to the current battery of tests provided by ImPACT and increase the reliability of detecting head impact-induced changes.

Eye movements have the potential to provide objective, interpretable, and mechanistic insight into traumatic brain injury. Eye tracking is non-invasive and little training is needed to collect eye tracking data. Prior work has shown that eye movements can be used to indicate clinically-relevant neuromotor and cognitive deficits (25–27), including those measured by ImPACT. As there is a physical link between the optic nerve and the eye (i.e., its physical connection), it stands to reason that there is also a link between increased ONSD (i.e., swelling of the optic nerve) and eye movements. Whereas, ImPACT scores can only offer diffuse perspectives on cognitive functioning, and ONSD captures a global metric of brain trauma as a proxy for ICP, eye movement features can point to specific neural pathways impacted by the injury. With distinct neural pathways governing visual, visual-to-motor, and motor processing functions (28, 29), as well as a range of discrete eye movements, such as saccades and smooth pursuits, deficits observed in eye movements can provide insights into anatomical phenotyping of the brain injury. Yet, explicit links between eye movements, ONSD, and ImPACT scores have not previously been demonstrated.

The focus of the present study is to investigate the potential for oculomotor features to provide graded indication of physiological changes, through a season of participation in contact sports, that may complement existing diagnostic tools, such as ONSD measurement and ImPACT scores. Rather than draw distinction between concussions and sub-concussive head impacts, both categories are included in the analysis to reflect head acceleration events of various magnitudes on the continuum (30). High school American football and soccer athletes were tracked through the course of a sports season. ImPACT scores, ultrasound ONSD, and eye tracking data were collected pre-season, twice during the season (early and late), and once post-season. Given the high risk of concussions and repeated sub-concussive head impacts associated with both soccer and football, our hypotheses were: (1) ONSD would increase and ImPACT scores would degrade through the season relative to baseline, and (2) oculomotor features would effectively predict both anatomical changes reflected by ONSD and neuro-functional status assessed via ImPACT. Absolute changes in physiological (i.e., ONSD) and behavioral (i.e., ImPACT) features were quantified, as well as those relative to the pre-season measurement. Oculomotor features were used to predict both the ImPACT scores and the ONSD measurements, and prediction accuracy reported. Finally, oculomotor features that were most predictive of ONSD and ImPACT scores were interpreted to gain a deeper understanding of neurophysiological deficits associated with high school soccer and football players over the course of a season. These insights could in turn facilitate novel mechanistic models, linking neural structures and oculomotor function, which can help to localize brain injury based on motor deficits and in turn guide interventions.

## 2. Data Collection

### 2.1. Subjects Enrollment

Twenty-four high school male football players and seven high school female soccer players, ages 15–18, were enrolled in the study which was conducted by Purdue University. Of those enrolled, complete pre-season, in-season, and post-season, data were collected for twenty-three of the football players and all seven soccer players. Pre-season and in-season activity involved the subjects' respective sport practices, drills, and games. Additionally, some subjects participated in other sports during the post-season assessment. All participants provided written informed consent and procedures were approved by both the Purdue Institutional Review Board and the MIT Committee on Use of Humans as Experimental Subjects.

### 2.2. Head Impact Data

The helmets of the 24 football players were equipped with the Head Impact Telemetry System (HITS; Simbex, LLC, Lebanon, New Hamshire). All recorded accelerometry traces were filtered based on procedures outlined in Cummiskey et al. (31). For each player, the number of hits per week, the cumulative number of hits, and the cumulative magnitude of the hits (accelerations measured in multiples of the gravitational constant [g]) were recorded. The number of head impacts was recorded to inform the discussion on the effects of repetitive sub-concussive head impacts (21). At the time these data were collected (2012), there was not a reliable method of affixing the HITS sensors to the heads of the soccer players via headbands. As such, HITS data was not present for the seven soccer players.

### 2.3. ImPACT Scores

In each testing session, subjects completed the computerized ImPACT. For the purposes of this study, ImPACT scores were used as an indicator of neurocognitive function and did not influence return-to-play decisions. Subjects were administered the online ImPACT, version 2.1, to monitor neurocognitive function for changes during the season. The ImPACT consists of six subtasks, including Verbal Memory, Visual Memory, Visual Motor, Reaction Time, Impulse Control, and Subjective Symptom Score. Six composite measures are calculated from summary statistics derived from one or more of these sub-tasks (32). The Verbal Memory composite includes word, symbol, and letter recall tests and provides an evaluation of attention, learning, and verbal memory. The Visual Memory composite consists of object recall tests to target visual attention, scanning, learning, and memory skills. The Visual Motor composite assesses visual processing, learning, memory, and motor response speed with metrics from the object and letter recall tests. The Reaction Time composite incorporates parameters from the object recall test as well as color and symbol matching tests to evaluate response speed. The Impulse Control composite evaluates the number of errors committed during the object recall and color match tests. This metric is used in the interpretation of other scores and overall test validity. The Symptom Score composite is a sum of values from a scaled set of concussion-related symptoms reported by the subject. The symptom score was omitted from our analysis based on the reliance of subjective information. We were interested in investigating changes in performance based on initial pre-season testing. Accordingly, scores were used in their raw rather than percentile form.

#### 2.3.1. Principal Component Analysis of ImPACT Scores

In the present data, six ImPACT component scores were found to be highly correlated, with correlations between the four cognitive composites (Verbal Memory, Visual Memory, Visual-Motor, and Reaction Time) ranging between 0.33 and 0.53. To avoid redundancy in fitting models due to these correlations, Principal Component (PC) Analysis was performed on the ImPACT scores prior to further analysis. Higher values on Verbal Memory, Visual Memory, and Visual-Motor scores correspond to better performance, while lower scores on the Reaction Time, Impulse Control, and Symptom Score correspond to better performance. To account for the reversed trends, the reciprocals of the latter three scores were used during calculation of the PCs. This way, larger values in individual composite scores denote better performance in the original space, and therefore, higher functional neurocognitive performance. In inverting the PCs to ImPACT composites, the first PC was found to have a weighting vector of [0.48, 0.49, 0.50, 0.51, −0.04, −0.09] , which demonstrates that the influence of Impulse Control and Symptom Score is negligible compared to the other four composites because the weights are about an order of magnitude lower than the rest. The resulting first PC was taken as a single latent factor that captures the sensorimotor and cognitive components of the ImPACT composites. This aggregate measure of the ImPACT score is referred to as “PC-ImPACT” throughout this manuscript. The first PC explained 60% of the variance in the ImPACT scores. Additional PCs of the ImPACT scores could be used as outcome measures in future work, but for simplicity of a single outcome variable in these machine learning procedures, just the most explanatory PC was used.

### 2.4. ONSD Measurements

**Figure 1:**
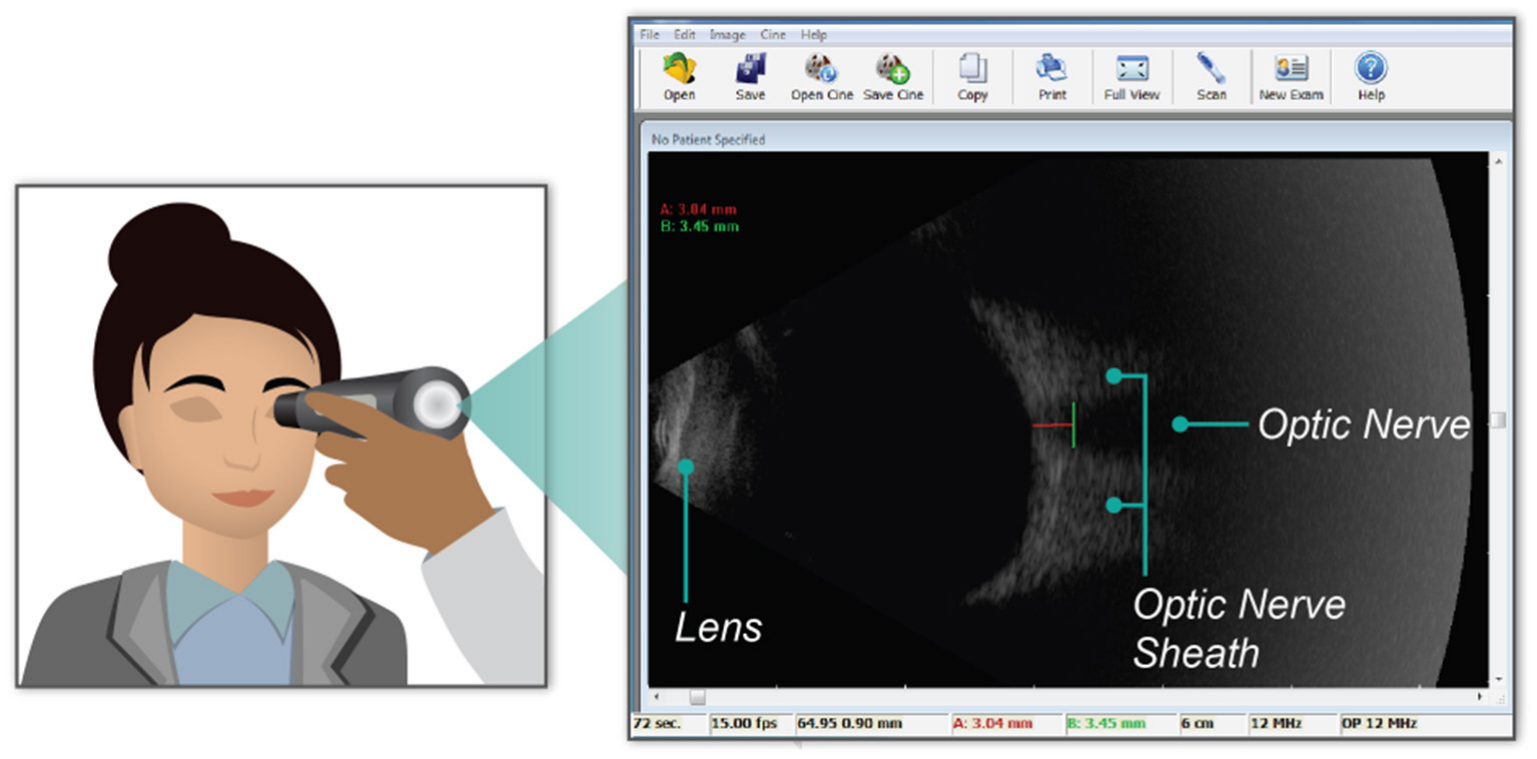
Graphical representation of the placement of the ultrasound probe over the subject's closed eye lid (*left*). The blowout image (*right*) shows a single frame within the video recorded during data collection. Key anatomical landmarks (i.e., the lens, optic nerve, and the optic nerve sheath) are labeled in the ultrasound image. Further, the green line on the ultrasound image shows the data measurement made of the optic nerve sheath diameter. In this example, the diameter is 3.45 mm.

An ophthalmic ultrasound probe, Interson model OP-12 MHz, was used to image the optic nerve sheath. The maximum scan depth of the probe is 6 cm and can be readily used to image the posterior eye and retina. Images were taken by pressing the probe gently into transduction fluid on the subject's shielded left eyelid and acquired with Interson software on a Windows laptop. A video of ~5 s in duration was recorded and stored. Measurements were taken 3 mm posterior of the globe and perpendicular to the central axis of the optic nerve. In Figure 1, an example image is shown. The red line shows the depth of measurement posterior to the globe, 3.04 mm in this case. The green line shows the ONSD measurement, which is 3.45 mm in this example. Of the 129 ocular ultrasound measurements collected from all subjects, a total of 33 tests were excluded due to poor image quality, and 96 measurements were retained. To minimize inter-operator interpretation variability, each measurement was validated by an independent reviewing team. All measurements were made by members of the research team that had received specific training in performing ONSD measurements.

### 2.5. Eye Tracking Data

**Figure 2:**
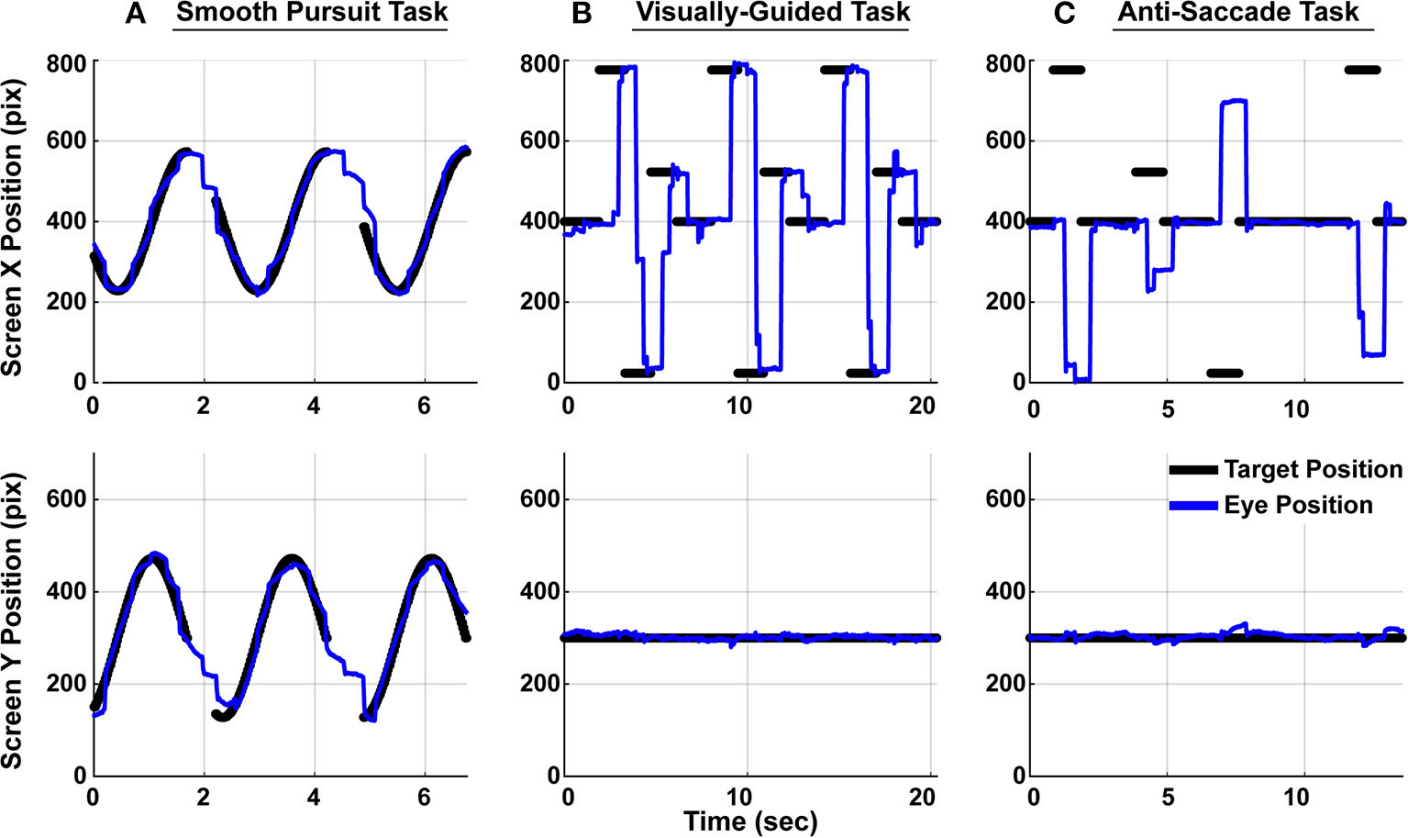
Subjects performed three eye tracking tasks. Target position is denoted with *black dots* and subject gaze position on screen is denoted as a *blue line*. The top panels show movements in the horizontal direction and the bottom panels show movement in the vertical direction. Only a portion of the full two-minute trials are shown here. **(A)** In the smooth pursuit task, a target traveled at constant speed in a circle around the screen. There were breaks in the target trajectory between cycles. **(B)** In the visually-guided task, a target jumped between prescribed position along the horizontal axis. Ten total unique positions were observed through the course of the whole trial. **(C)** In the anti-saccade task, subjects were instructed to make a saccade to the equal and opposite position on the horizontal axis from the target position.

Monocular eye movement data were recorded using an SR Research Eyelink1000 eye tracker. All eye movement data was collected and analyzed at 1,000 Hz. While their heads were supported with a chin rest 60 cm away from the monitor, subjects' left eye was tracked as they performed three eye tracking tasks, as shown in Figure 2. Eye position calibration was performed first within the SR Research software and met the standard for high-quality calibration before proceeding to the eye tracking tasks, detailed below. Each subject performed one trial of each task.

#### 2.5.1. Smooth Pursuit Task

Subjects were instructed to track a target that moved in a circular pattern around the screen. The target appeared on the screen for roughly 1.5 s followed by a 0.5 s break before reappearing (Figure 2A). The target made 35 cycles in the Smooth Pursuit task, which lasted just over 2 min.

#### 2.5.2. Visually-Guided Saccade Task

Subjects were instructed to accurately and quickly make eye movements to the target position on the screen. In this task, the target instantaneously jumped from one of ten unique locations to another. Each peripheral target appeared on the screen for 1.5 s and each central target appeared for 2 s. In total, the target jumped 78 times within a single trial, which lasted 155 s. This task is depicted in Figure 2B. In the figure, only a subset of the locations are visualized for graphical clarity.

#### 2.5.3. Anti-saccade Task

As subjects fixated on the central fixation dot, a target appeared in the periphery. Subjects were instructed to suppress the reflex to look at the target and instead, make a saccade to the equal and opposite location on the screen (Figure 2C). A saccade made to the target in this anti-saccade task was considered an error, but no online feedback was provided to subjects regarding their accuracy. Targets appeared in the periphery for 1 s each, and the presentation of the central fixation duration, randomly drawn prior to the study, could be 1, 2, 3, or 4 s. There were a total of 18 peripheral targets (nine on each side) in a trial, which lasted 90 s. Every subject saw the same target positions and associated durations, though the movements would have appeared random to them. In the interest of maximizing the quantity of data in the limited time available to perform the recordings, the targets only moved in the horizontal dimension during the visually-guided and anti-saccade tasks.

### 2.6. Time Points of Measurements

The ImPACT, ONSD, and Eye Tracking measurements were made at the same time, so the dates for the three modalities are the same. For each subject, the average (+/− SD) time between measurements was 28 ± 9 days (Pre-to-Early Season), 38 ± 8 days (Early-to-Late Season), and 33 ± 12 days (Late-to-Post Season). The means of the measurement dates across subjects were 28 July 2012, 25 Aug 2012, 02 Oct 2012, and 07 Nov 2012, respectively, for the Pre-, Early-, Late-, and Post-Season measurements. Of the 96 total measurements used (cf. section 2.4), 30 corresponded to Pre-Season, 30 corresponded to Early-Season, 28 corresponded to Late-Season, and eight corresponded to Post-Season.

## 3. Methods

### 3.1. Computing Oculomotor Features

The oculomotor features, calculated from the eye tracking data, were aimed at characterizing visual-motor processing (33), motor control (34), and cognitive function (35). To evaluate visual-motor processing, metrics, such as reaction time of saccades in response to step changes in target position, velocity gains of smooth pursuit tracking, and the number of catch-up saccades that occurred during smooth pursuit were computed. To quantify motor control, metrics, such as fixation dispersion, ratio of the peak velocity to the mean velocity of the saccade main sequence, and the accuracy of initial saccades to targets were computed. Finally, to quantify cognitive function, features, such as errors on the anti-saccade task and the number of fixations prematurely broken were used. A full list of oculomotor features, their description, and the putative sensorimotor or cognitive association is listed in Supplementary Table 1. Features were computed in MATLab (Mathworks Inc., Natick, USA).

### 3.2. Mixed-Effects Modeling

A predictive modeling analysis using Linear Mixed-Effects (LME) regression was conducted to investigate whether oculomotor features can serve as a reliable indicator of ONSD or ImPACT scores. Model design was guided by the longitudinal, repeated-measures nature of the study. Repeated physiological and cognitive measurements obtained from the same individual suggests an inherent relationship between them, via latent within-subject factors of potential relevance, and therefore cannot be assumed to be independent from each other. These relationships may vary differently across individuals under the same conditions (36).

To account for these latent within-subject factors, LME models contain separate terms to capture between-subject variability at the population level, as well as within-subject variability at the individual level. The former is commonly denoted as “fixed effects,” as it corresponds to relationships that remain constant for all subjects in the population. The latter represents the within-subject variability as the outcome of a combination of latent random variables, and is thus denoted as the “random effects” component of an LME model. Mathematically, an LME model can be represented as:

(1) $$\begin{array}{l} y=X\beta +Zu+\epsilon \end{array}$$

where

$$\begin{array}{l} \text{y}=\text{known vector of observed outcomes}\\ \text{X}=\text{known matrix of fixed effects values in the data}\\ \text{Z}=\text{known matrix of random effects values}\\ \beta =\text{unknown vector of fixed effects coefficients}\\ \text{u}=\text{unknown vector of random effects coefficients}\\ \epsilon =\text{unknown vector of random errors} \end{array}$$

Oculomotor features are applied as the fixed effects matrix X (see section 3.2.2), and subject identifiers as random effects Z. Separate models apply ONSD measurements and ImPACT scores, respectively, as the observed outcome vector *y*. The fixed effects coefficients β, random effects coefficients *u*, and random errors ϵ are computed using Maximum Likelihood Estimation to optimize the fit of the statistical model to the observed data.

#### 3.2.1. Two Model Variants

In application to the current study, two LME model variants are explored: raw (R-LME), and subtractive-normalized (SN-LME). R-LME seeks to identify the degree to which raw oculomotor features are predictive of neurophysiological state (raw ONSD or ImPACT scores). Both raw ONSD and raw ImPACT scores are valued clinically as simple, interpretable measures of neurological status, with raw ONSD indicating severity for concussions and ICP (14) and raw ImPACT scores indicating cognitive and functional state. Thus, a direct predictive relationship would demonstrate the potential of oculomotor features to be harnessed as a resource for clinical assessment that can provide deeper insights about the nature of the physiological changes observed.

Due to the natural variability in baseline cognition and physiology across subjects, the SN-LME modeling approach was used in order to normalize for these differences, allowing for assessment of changes through the sport season that can be meaningfully compared across subjects. Specifically, the SN-LME model investigates whether change in oculomotor features can be used as an objective and robust indicator of changes in ICP. This model predicts change in ONSD or ImPACT scores throughout the season based on change in oculomotor features. Change in both predictors and outcomes is computed by subtractive normalization relative to each subject's preseason baseline.

#### 3.2.2. Modeling Setup

Performance of the LME models was evaluated using a Leave-One-Session-Out (LOSO) Cross-Validation methodology. Within a given iteration of training/testing, one data point (“session”) from one subject was held out for testing, while the rest of the sessions were retained for training the model. This process was repeated, each time holding out a different session for testing, such that all sessions serve as a test case once. Therefore, in each iteration, the held out data point was the testing set and the rest of the data constituted the training set. In the SN-LME variant, first sessions (corresponding to baseline preseason measurements) are only used for normalization and thereafter excluded from model fitting. Metrics to evaluate prediction accuracy consist of Pearson correlation between predicted and actual outcomes, as well as root mean squared error (RMSE) and R-squared. Significance of results was determined using *p*-value at the 0.05 level.

For each LME model, subject identifiers were applied directly as the random effects. Prior to application as fixed effects, oculomotor features were first transformed via principal components analysis (PCA) to a lower-dimensional space. An optimal number of principal components was dynamically derived for each new model built within the LOSO cross-validation methodology described above. Within each iteration of LOSO, an optimization procedure was applied to the current training set (all but the one left-out session) in order to find the number of PCs that maximizes prediction accuracy. An inner loop of LOSO cross-validation was run only on this current training set, and Pearson correlation of predicted vs. actual outcomes from the inner loop was evaluated across a brute force enumeration of different numbers of PCs. The number of top PCs yielding the maximum correlation between predicted and actual in the inner LOSO loop informed the selection of PCs as the fixed effects predictors for the model in the main outer LOSO loop.

For the SN-LME model, the oculomotor features were subtractive normalized prior to PCA. Likewise, subtractive normalized PC-ImPACT (section 2.3.1) was computed by subtractive normalizing individual raw ImPACT scores prior to PCA.

## 4. Results

### 4.1. Head Acceleration Events

**Figure 3:**
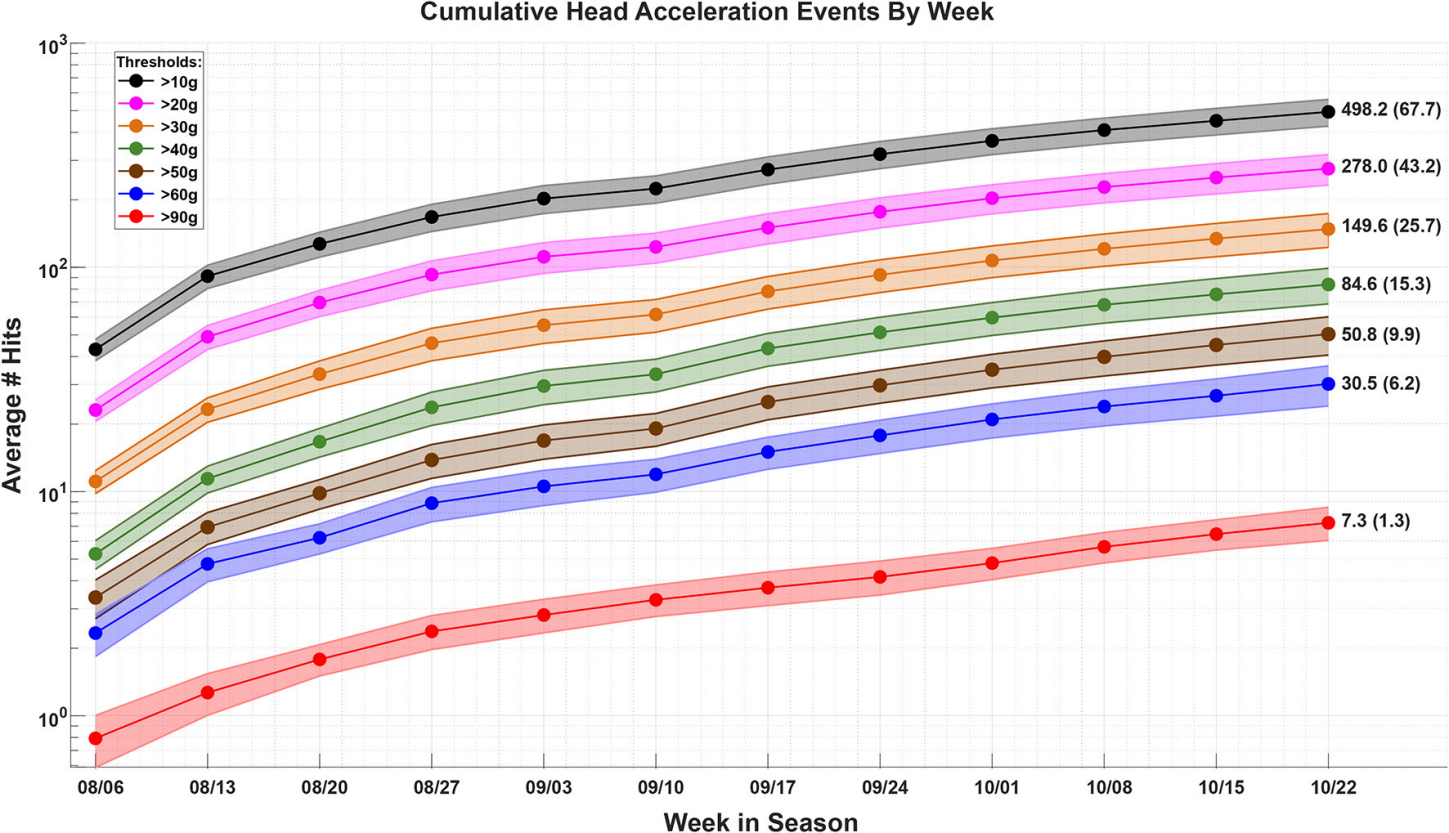
The average (±stdev) number of head acceleration events (HAEs) sustained across the football player cohort, within each week, through the course of the season. The HAEs are thresholded by levels of acceleration, as measured with the Head Impact Telemetry System (HITS). As there are many more HAEs ≥10 g as there are ≥90 g, the data are plotted on a log scale.

The number of head acceleration events (HAEs) were recorded each week for each football player. The HAEs were thresholded based on the peak linear acceleration (31, 37). The average number of HAEs across the football cohort, thresholded by levels of acceleration, are plotted as a function of week during the season Figure 3. The players enrolled in this study were consistently involved with the sport through the course of their season and there was a steady increase in the number of HAEs through this time. While Figure 3 shows that there was head contact, the overall trends in the figure are not representative of an individual's exposure profile. The correlation between the number of HAEs and ONSD showed insignificant relationships (Supplementary Figure 1). While the HITS data provide reasonable measures of population-level HAEs, the estimates of individual exposure within short windows of time are noisy, potentially limiting the results of the HAE and ONSD correlations (31). Therefore, the HAE data are hereafter not used as an independent variable to predict physiological changes, rather, the relationships between the physiological variables are modeled.

### 4.2. ONSD and ImPACT Trends Through Season

**Figure 4:**
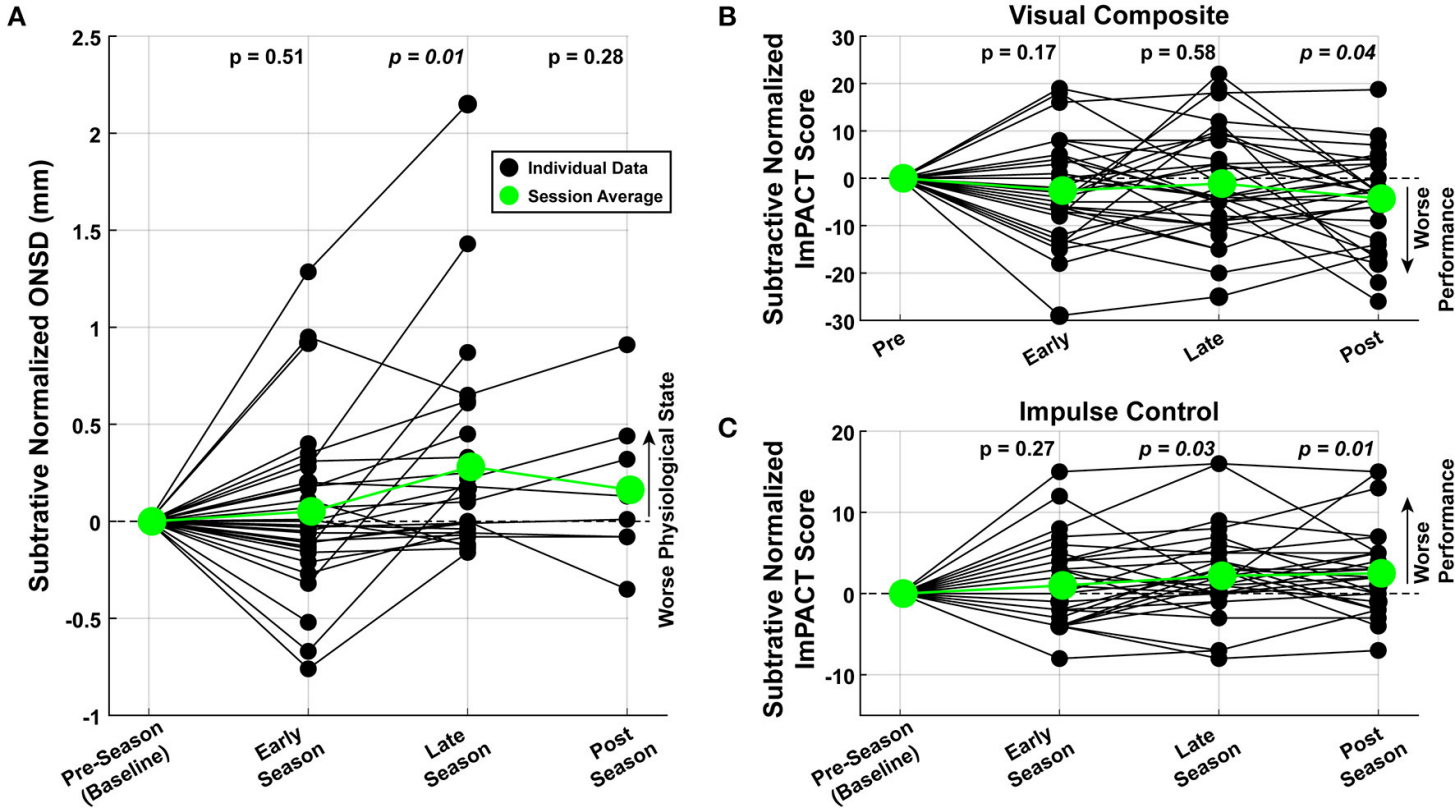
Graded changes in neurophysiology through the season. Measured values were normalized to the first recording for each subject. Each data point *(filled black circles)* represents a single measurement. The average across the subjects are plotted in green *(large filled circles)*. *t*-Tests performed with data from each session shows that, at the population-level, there was a significant **(A)** increase in ONSD, **(B)** decrease in ImPACT Visual scores, and **(C)** increase in ImPACT Impulse Control scores.

To highlight raw and relative changes that might have occurred through the course of the season, each subject's data were subtractive-normalized to their baseline measurement, collected pre-season. All 96 observations are shown in Figure 4 for normalized ONSD and two example ImPACT Score Composites. As a population, there was a significant increase in ONSD toward the end of the season (*t*-test, *p* = 0.01), significant decrease in the ImPACT Visual Composite score (*t*-test, *p* < 0.05), and significant increase in the ImPACT Impulse Control score (*t*-test, *p* < 0.05), all of which indicates the presence of neurophysiological changes.

For the ImPACT scores and ONSD values, Cohen's *d* values were computed comparing the football (Male) and soccer (Female) cohorts. None of the Raw outcome variables showed large effect sizes (i.e., none were above 0.8) and only the ImPACT Verbal score (Cohen's *d* = 0.64; larger values for Soccer players) showed a medium effect size. The other ImPACT scores, the ONSD values, and the subtractive-normalized versions of the variables, all showed low or negligible effect sizes.

### 4.3. Correlation Between ONSD and ImPACT Scores

**Figure 5:**
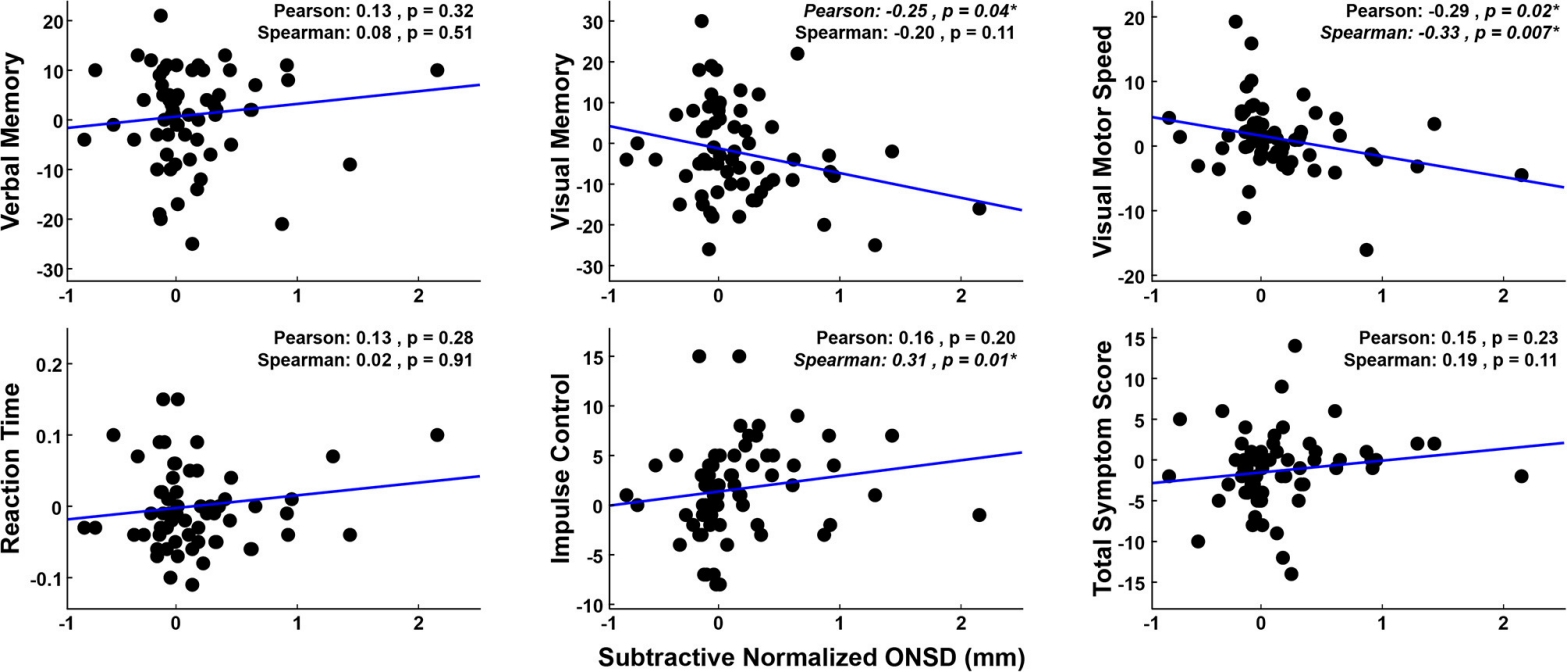
Subtractive-normalized ONSD values are plotted against subtractive-normalized ImPACT composite scores. Data used for within-subject normalization [i.e., values at (0, 0)] are not plotted or used in correlations. Changes in the Visual Motor, Visual Memory, and Impulse Control scores show significant correlations with the changes in the ONSD values, in directions that link declining ImPACT performance with declining physiological (ONSD) state.

The relationship between increased ONSD and decreased performance on the ImPACT (cf. Figure 4) may be linear or non-linear but monotonic. To elucidate linear relationships, Pearson correlations were performed, and to identify potential non-linear, yet monotonic relationships, Spearman correlations were also performed. For completeness, both correlation coefficients, as well as their respective *p*-values are reported in Figure 5. There are significant correlations between ONSD values and the Visual Memory (*Pearson*), Visual-Motor (*Spearman*), and Impulse Control (*Spearman*) ImPACT composite scores. To determine whether outlier data points were driving the linear model fits, a Cook's D analysis was run for each fit shown in Figure 5. There were no data points that met the criterion of having Cook's D values >1 (38), indicating that no individual outlier data points were unduly driving the results. These trends observed in ImPACT scores (decreased Visual, decreased Visual-Motor, and increased Impulse Control scores) linked to increased ONSD values are congruent with the expectation that head impact exposure leads to increased ONSD and decreased performance on the ImPACT (18, 19). Increased head exposure, increased ONSD, and decreased performance on the ImPACT are coincident and correlational in the present analyses. Additional research is needed to show the causal relationships between head exposure and the observed physiological changes.

**Figure 6:**
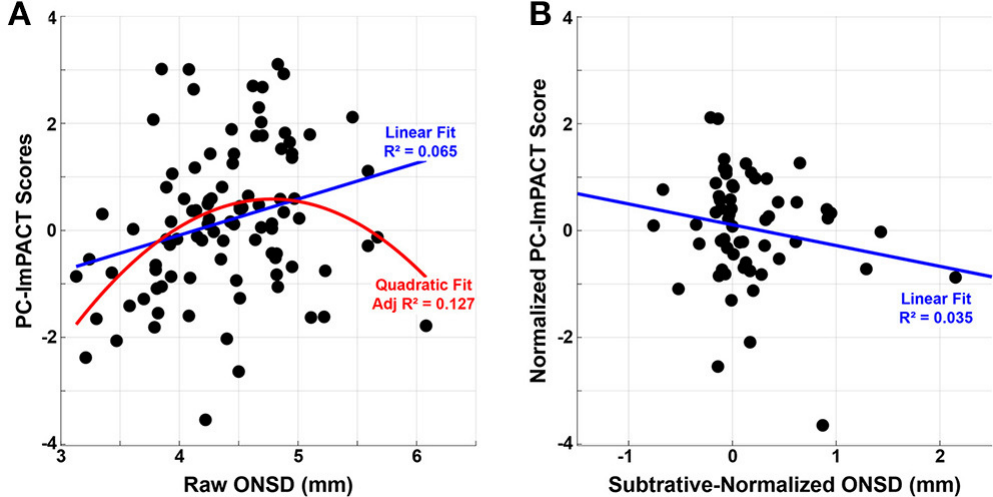
ONSD measurements correlate with the first principal component of the ImPACT scores (PC-ImPACT). **(A)** Raw ONSD measurements show a significantly positive relationship with PC-ImPACT scores with a linear model (*p* < 0.01). A quadratic fit modeled the data better (*p* < 0.001), showing a higher adj *R*^2^. **(B)** Normalized ONSD and PC-ImPACT scores show a negative relationship, though the trend is not statistically significant (*p* = 0.154).

In clinical settings, an ONSD measurement >5 mm would indicate an elevated ICP value (39). Accordingly, raised ONSD values would correspond to a decrement in ImPACT performance (i.e., lower PC-ImPACT values, which is the first principle component of the ImPACT scores). To describe the relationship between ONSD measurement and PC-ImPACT scores, the two variables are plotted in Figure 6A. A linear fit to the data shows a significantly positive trend (*p* < 0.01). However, a quadratic fit to the data, also statistically significant (*p* < 0.001), has a higher adjusted R^2^ value, indicating a better characterization of the data. The better fit using a quadratic model points to a non-linear relationship between the ONSD value and PC-ImPACT scores. The decreased PC-ImPACT score for high values of ONSD (>5 mm) are expected from the clinical literature. However, the positive relationship when including all ONSD values, not just the above-threshold values, is a new finding. The quadratic model points to an interplay between positive correlation between the variables at lower values of ONSD, but a negative correlation at higher values of ONSD (e.g., clinical threshold of >5 mm). Our prediction of the changes in neurophysiological variables was that increased ONSD would be coincident with decreased performance on the ImPACT. In Figure 6B, the normalized variables show such a trend, but the slope is not statistically significant at the (*p* < 0.05) level.

### 4.4. Predicting ONSD and ImPACT Scores From Oculomotor Features

As previously mentioned, ONSD provides an indication of an individual's physical, physiological state, while ImPACT scores reflect neuropsychological status of the individual. Oculomotor features, derived from eye tracking tasks, have the potential to be predictive of both the anatomical and functional states of the individual. In the following sections, oculomotor features are used to predict both the ONSD and PC-ImPACT scores. The model is then investigated to understand the specific features that are most predictive. To evaluate the performance of the model, the model-predicted values are plotted against the actual values. The correlation, root mean squared error (RMSE), bias (mean of predicted − mean of actual), and *p*-value are reported. Note that both the RMSE and bias are in the units of the outcome measure, thereby providing direct interpretation of the performance of the model. Model performance is always evaluated on the prediction of held-out data.

#### 4.4.1. Predicting ONSD

**Figure 7:**
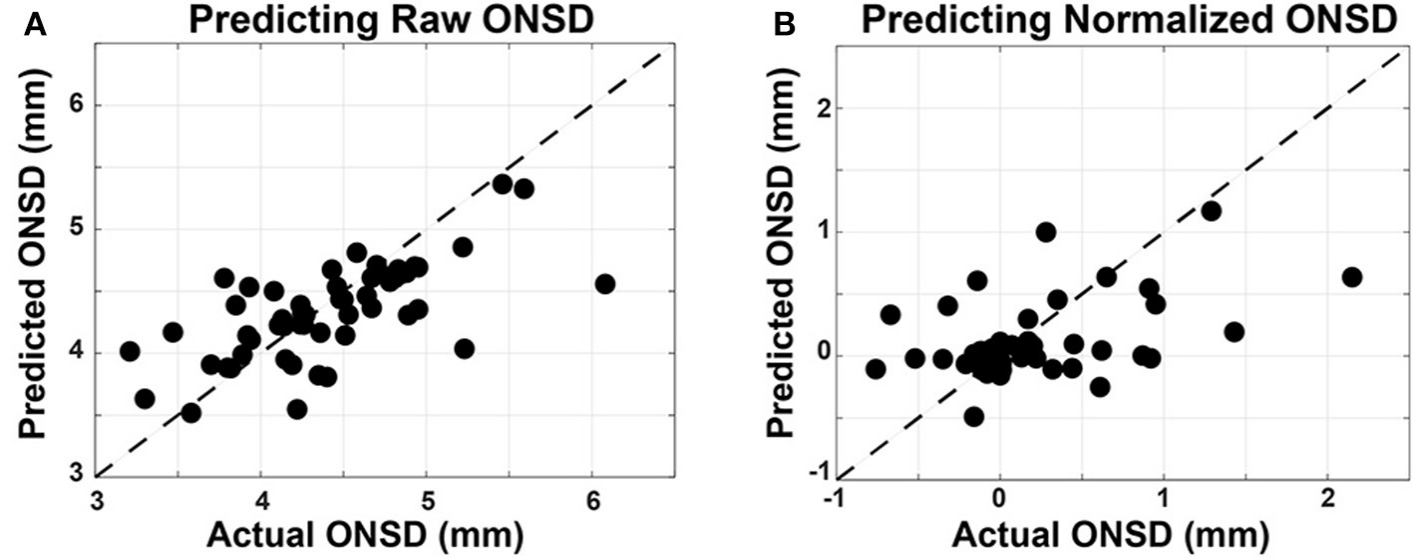
Linear mixed-effects models used to predict ONSD measurements from oculomotor features. Oculomotor features are used as fixed effects and the subject ID is used as a random effect in the LME models. Measured ONSD values are plotted on the horizontal axis and the ONSD values from the model, predicted on held out data, are plotted on the vertical axis. The unity line is plotted with dashes. **(A)** In predicting raw ONSD values, the R-LME model had measures of performance as such: Corr = 0.698, RMSE = 0.417 mm, and Bias = 0.27 mm. **(B)** In predicting the subtractive normalized ONSD values, the SN-LME model had measures of performance as such: Corr = 0.451, RMSE = 0.482 mm, Bias = 0.12 mm).

As described in section 3.2, Linear Mixed Effects (LME) modeling was used to predict both the raw and normalized ONSD values, referred to as the R-LME and SN-LME models, respectively. Given the clinical significance of ONSD thresholds in assessment of ICP for diagnosis of brain injury, the R-LME model examines the degree to which raw oculomotor features can predict raw ONSD (Figure 7A). The SN-LME model uses change in oculomotor features to predict changes in ONSD through the sport season (Figure 7B). In the SN-LME model, the changes are computed using subtractive normalization of both the predictors and outcome values. A Cook's D analysis showed that no data points had distance >1, indicating that no outlier data points were driving the model fit.

These results show a significant (*p* < 0.001) capability for accurately predicting the raw and normalized ONSD values from oculomotor features. RMSE values, indicative of the variance about model estimates, are reported in the units of the outcome variable. Therefore, that measure of model performance is directly applicable to clinical settings wherein the absolute value of the ONSD is of diagnostic value. When predicting the subtractive-normalized ONSD values, this model shows capability to determine a within-subject change in ONSD value. This could be applied as a screening tool to determine if there are subtle increases in ONSD, and therefore ICP. This could be applied as a screening tool to determine if there are subtle increases in ONSD, and therefore ICP, over the course of a sports season. Furthermore, future work that quantifies repetitive head acceleration events in a similar population, in combination with the currently presented methodology, may provide meaningful insight into suggested connections between these objective assessment modalities and repetitive sub-concussive impacts.

#### 4.4.2. Predicting ImPACT Scores

R-LME and SN-LME models were also built to predict PC-ImPACT scores, with prediction accuracy evaluated on held-out data using a Leave-One-Session-Out paradigm (see 3.2.1. Model performance in predicting both raw and normalized PC-ImPACT scores were both significant (*p* < 0.001).

**Figure 8:**
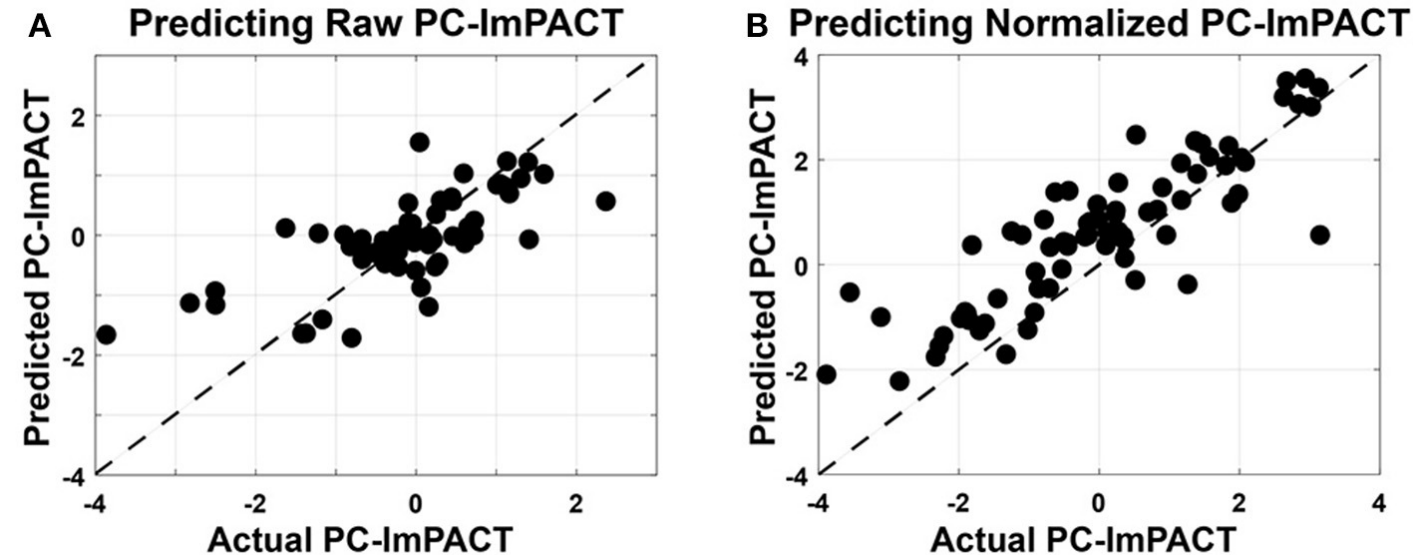
Linear mixed-effects models used to predict PC-ImPACT scores from oculomotor features. Oculomotor features are used as fixed effects and the subject ID is used as a random effect in the LME models. Measured PC-ImPACT values are plotted on the horizontal axis and the PC-ImPACT values from the model, predicted on held out data, are plotted on the vertical axis. The unity line is plotted with dashes. **(A)** Predicting the raw PC-ImPACT scores using the raw oculomotor features had high positive correlation (Corr = 0.86), but also higher RMSE (1.030), due to the bias (1.08) in the model. **(B)** When predicting the subtractive normalized PC-ImPACT scores, the correlation performance only dropped slightly (Corr = 0.712), but the RMSE (0.744) was also lower along with the bias (0.52).

In predicting raw PC-ImPACT scores using raw oculomotor features, R-LME showed significant correlation and predictive power (Corr = 0.86, RMSE = 1.03, Bias = 1.08) (Figure 8A). A Cook's D analysis showed that no data points had distance >1, indicating that no outlier data points were driving the model fit. The higher RMSE can be attributed to the presence of a bias, causing an offset in the prediction. These results show that oculomotor features can be a rich source of screening information capturing a wide range of neurocognitive function. In addition to reducing the possibility of over-fitting, the process of performing the PC analysis on the ImPACT scores provided a useful representation of the overall performance across the ImPACT.

In predicting the SN PC-ImPACT scores from SN oculomotor features (Figure 8B), the performance of the SN-LME model had lower correlation, but also lower RMSE, in part due to the low bias (Corr = 0.712, RMSE = 0.74, Bias = 0.52). It is expected that predicting changes in ImPACT scores is inherently more challenging than predicting raw values, because the dynamic range of the outcome variable is reduced and the majority of the data are clustered at zero. Therefore, predictions by the SN-LME model are much more susceptible to noise than with R-LME. Additionally, the R-LME model can leverage the inter-subject variability present in the individualized offsets of values, but with those removed during subtractive normalization, the inter-subject variability is reduced, leaving only intra-subject variability in PC-ImPACT changes. Nevertheless, SN-LME showed a significant and positive relationship between predicted and actual subtractive-normalized PC-ImPACT values. ImPACT scores are used as a continuous measure of functional performance. Therefore, the capability to use oculomotor features to predict relative changes in PC-ImPACT scores could provide new and useful screening value.

#### 4.4.3. Importance of Mixed Effects Modeling

To predict ONSD and PC-ImPACT scores from oculomotor features, a linear mixed effects model was used. As a point of comparison, Table 1 shows the measures of performance when subject ID is used as a random effect variable or when only a fixed effect variable is used (i.e., standard regression model). Results from the models that include subject ID as a random effects variable have been detailed in Figures 7, 8. As compared to the mixed effects model, the standard regression model performs much worse. First, the models predicting ONSD are not statistically significant, show correlation values close to zero, and have higher RMSE. Though the models predicting PC-ImPACT scores are statistically significant, the correlation values are much lower and again, have higher RMSE values. The comparison between these two methods of modeling the results show the importance of taking the within-subjects effects into account and therefore support LME modeling.

**Table 1:** Comparing the performance of LME models with standard regression models.

			**ONSD**				**ImPACT**			
**Model**	**Fixed Effects**	**RandomEffects**	**Corr**	***p*****-Value**	**Bias**	**RMSE**	**Corr**	***p*****-Value**	**Bias**	**RMSE**
		Subject	0.698	2.2e-09	0.27	0.417	0.860	1.8e-22	1.08	1.030
R-LME	OculomotorPCs	None	0.030	0.830	0.01	0.646	0.258	0.03	0.03	1.612
		Subject	0.451	0.001	0.12	0.482	0.712	2.8e-11	0.52	0.744
SN-LME	OculomotorPCs	None	0.058	0.690	0.01	0.560	0.489	3.5e-05	0.19	0.931

*Without subject ID as a random effect, the LME models reduce to a standard regression model. The performance with a mixed effects design is much higher than a standard regression model, indicating the value added by incorporating the within-subject effects*.

#### 4.4.4. Relative Contribution of Oculomotor Components

As described in section 3.2, the fixed effects in the LME model design included PCA-transformed components of the oculomotor features, selected through a cross-validation process, and an additional intercept term for the R-LME models. On principle, an intercept term was not added to the SN-LME models as the data were subtractive normalized and therefore, already without offsets.

Toward assessing the scale of variance in the oculomotor features that contributes most to the predictive performance of the LME models, Table 2 shows the mean fixed-effects regression coefficient assigned to each oculomotor PC included in the models for each outcome type. The higher the coefficient, the greater the contribution of that variable. Note, coefficients for the subject ID term are not listed in the table, as they are part of the random effects of the LME models. In predicting ONSD, only the first PC was needed, indicating that there is sufficiently high information content in the first PC such that additional PCs did not add predictive capability. For PC-ImPACT models, up to five oculomotor PCs were used. Due to the nature of PCA, the largest percentage of variance in the data is captured by the first PC, with subsequent PCs representing increasingly smaller scales of oculomotor variance.

**Table 2:** Contributions of PCA-transformed oculomotor features fixed effect predictors to the LME model.

**Fixed Effects**	**Predicting Raw ONSD**	**Predicting Raw PC-ImPACT**	**Predicting SN ONSD**	**Predicting SN PC-ImPACT**
Intercept	4.366 (0.006)	0.024 (0.014)	–	–
Oculomotor PC-1	0.005 (0.002)	0.018 (0.004)	0.018 (0.003)	0.029 (0.004)
Oculomotor PC-2	–	0.039 (0.011)	–	0.045 (0.004)
Oculomotor PC-3	–	0.156 (0.014)	–	0.155 (0.035)
Oculomotor PC-4	–	0.018 (0.014)	–	–
Oculomotor PC-5	–	0.099 (0.007)	–	–

*Weights averaged across LOSO models, and their standard deviations in parentheses, are reported for each PC. The number of PCs to be used was determined through LOSO cross-validation (see 3.2) and therefore, some models had fewer than five PCs. An intercept term was not included in the SN-LME models on the principle that data already had offsets removed by normalizations. PCs representing subtler variations in the oculomotor feature space (notably, PC-3) are observed to be more informative in predicting ONSD and ImPACT score outcomes*.

These results reveal a salience of subtler variances in the oculomotor feature space to predicting PC-ImPACT scores. The relatively lower average of LME coefficients for PC-1 shows that it is not the most informative for PC-ImPACT prediction, despite representing the most variance in the oculomotor features. In contrast, higher weights are assigned by LME regression to PC-3 across both Raw and SN PC-ImPACT models. The smaller variations captured by PC-3 are therefore shown to be more important in capturing the physiological and functional changes given by ImPACT scores.

#### 4.4.5. Weighting of Oculomotor Features

**Figure 9:**
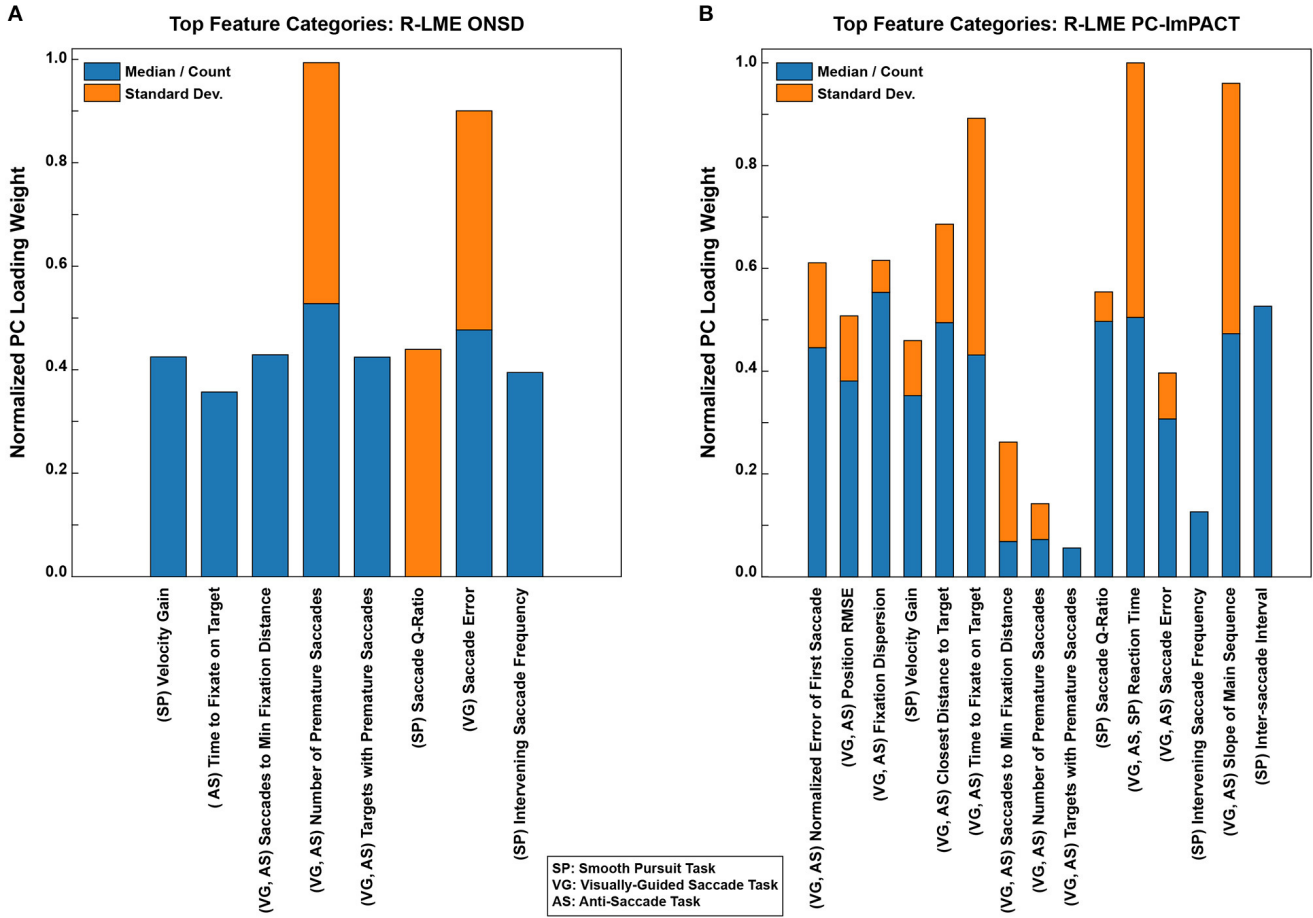
Oculomotor features that are predictive of ONSD and ImPACT scores. The abscissa represent the relative loading weights of the features on the PCs. The higher the loading, the greater the impact on the PC and therefore, the greater the contribution to predicting the outcome variable. The feature types are separated by whether they denote a median or count (*blue*), or a standard deviation (*Standard Dev.; orange*). The parenthetical abbreviation denote the tasks in which the features were computed from. Key features, proven to be informative at predicting both outcomes, are related to motor control and visual-to-motor processing.

The oculomotor features were selected to reflect visual, motor, and visual-to-motor processing within the eye tracking tasks. Though the PCs of oculomotor features were used in the LME models, the relative contributions of each feature to the PCs can be quantified to determine which features were more informative in the model. Within each test-and-train iteration, the top 20 features within each PC were grouped and scaled by the regression coefficient of that PC. The top features were selected using the magnitude of the loading onto PCs. The average loading was computed across all iterations and the important features used in predicting ONSD are shown in Figure 9A and similarly, the important features used in predicting PC-ImPACT scores are shown in Figure 9B. The higher the weight, the more the contribution of that feature. Within each prediction task, the weighting is normalized to the largest feature contribution.

Both models showed similarity in the features that were most predictive of the outcome variable. The three highest features in predicting the ONSD values were the total number of premature saccades made, the accuracy of saccades, and the saccade Q-ratio (peak velocity × duration/saccade amplitude). The Q-ratio is a strictly motor-relevant feature, which may be impacted by physical changes in the ONSD. The accuracy of saccades may reflect the visual-to-motor transformation. Finally, the number of premature saccades made could indicate more cognitive declines as the ability to inhibit saccade reflexes worsens. In predicting the PC-ImPACT scores, the reaction time, slope of the saccadic main sequence (i.e., saccade amplitude vs. duration relationship), and time to fixate on the target were the three most important features. The slope of the main sequence, relevant in all the tasks, reflects the motor component of saccades (i.e., amplitude, duration, and velocity relationship). The reaction time and the time to fixate on the target both reflect the visual-to-motor processing loop. In aggregate, these features represent the visual-motor processing loop of identifying a peripheral target, estimating its distance, and executing a saccade to that target. Optimal behavior would be to execute a single saccade to the target. However, due to functional or physical deficits, more saccades would have to be taken to foveate the target.

In sustaining repetitive head impacts, an individual might show relatively worse performance, measured as changes in the medians/counts of features, or they might show more variable performance, by increases in standard deviations of features. To tease apart the two outcomes, the medians or counts, and standard deviations of each measure were used as independent features for the models. The relative weightings of the medians or counts (Figure 9, *blue bars*) are shown in comparison to the standard deviations (Figure 9, *orange bars*). On average, individuals show more of a change in the medians/counts performance. Nevertheless, variability in oculomotor control also appears to be highly informative, especially for the key features that seem to be most informative.

## 5. Discussion

Eye tracking data, ONSD measurements, and ImPACT scores of high school American football and soccer athletes were collected through the course of a sports season. ImPACT scores and ONSD measurements each showed decline through the course of the season. Oculomotor features, computed from eye tracking tasks, were highly predictive of both ImPACT scores and ONSD. A linear mixed effects (LME) model, which accounts for a global relationship across subjects, as well as a subject-specific relationship between the oculomotor features and ImPACT scores or ONSD, proved to be crucial in generating a predictive model, highlighting the importance of considering individual variability in physiological responses. Future work is needed to causally link the head exposure to physiological changes observed through the course of the season. With such causal links in place, features of eye movements, as computed in the present work, can be used to provide additional insight into head impact-induced neurophysiological changes, which can guide subsequent rehabilitation protocols.

### 5.1. Clinical Relevance of Repetitive Sub-concussive Head Impact Exposure

The diagnostic criteria for head injury are discretized by levels of severity, labeled as mild, moderate, or severe (40). The current methods for clinical diagnosis do not capture the subtle, but cumulative, effects of repetitive, sub-concussive head impacts. Recent research has pointed to the neurological deficits of sub-concussive head insults (41–45) but the current standards of concussion detection might not be sensitive enough to detect those subtle changes (46, 47). The focus of this study was on the physiological changes that occur through the course of time for contact sport athletes. Future work quantifying the HAE of contact sport athletes, while implementing the presented framework, has value in bridging the physiological changes observed in the current work with documented individual athlete HAE.

In clinical settings, ONSD has typically been regarded as providing a binary report of the state of ICP (15, 48). Either the value is below a threshold, indicating a healthy state, or it is above, indicating injury (49, 50). However, this binary approach does not elucidate graded changes relative to baseline or the longer-term physiological consequences of repetitive head impacts. For example, evidence from long-duration spaceflights show that in microgravity, small increases in ICP and intraocular pressure, sustained over a long period, leads to irreversible damage to the eye and brain (51–53). In relation to ImPACT scores, which are currently used a relative measure of neurocognitive function, the relative changes in ONSD values show significant correlation with changes in ImPACT scores. The results of the Cook's D analysis showed that no individual data points were outliers in the model fit. This is especially noteworthy given the two data points following medically-diagnosed concussions. That they were not flagged as outliers indicates that the information in those data points can be captured using the same model as that used to quantify the effect of repetitive, sub-concussive head exposure. No unique exceptions need to be made for the concussion case vs. the sub-concussive cases.

### 5.2. Capability Provided by Eye Movements

While ONSD and ImPACT scores provide insight into neurophysiological function, they each have their limitations. Eye tracking has the potential to replicate the same information gained from ONSD and ImPACT and further provide mechanistic insight into the severity and sub-type of the injury. ONSD provides a measurement of the physical changes following head injury, but it requires a trained expert to administer the recording and analyze the result. Even with expert analysts, there can be some amount of variability in the reported value. ImPACT scores provide insight into neurocognitive function, but the results are non-specific to brain regions and the testing results are subject to the effort put forth by the individual.

Eye tracking studies can be performed by anybody and provide deterministic results, as compared to subjective readings of ultrasound images. The eye tracking modality can be combined with additional physiological modalities, such as speech (45, 54) and gait (47, 55), to improve the sensitivity of detecting injury-induced effects. Eye movements also have the potential to capture more information than just what is measured by ONSD and ImPACT scores. Given that the visual-motor processing involved with generating eye movements spans a large extent of the brain (29, 56, 57) and mechanistic models have linked functional behavior to anatomical substrates (58–60), motor dysfunction detected through eye movements could in turn inform about specific regions of injury and the potential functional sub-type of that injury. This can be useful in guiding subsequent rehabilitative therapy.

### 5.3. Importance of Subject Normalization

Within-subject normalization was meaningful in modeling the relationship between oculomotor features and the related neurophysiology, through the use of subtractive normalization and Linear Mixed Effects modeling. Subtractive normalization of oculomotor features to predict change in ONSD and ImPACT outcomes enabled the analysis and prediction of relative change. Because ONSD is correlated with eyeball transverse diameter, baseline non-pathologic ONSD varies from person to person, despite normal ICP (61). This makes it difficult to assess raw ONSD values directly, beyond a global threshold of >5 mm as an indicator of pathology. A meta-analysis, review ONSD values across many ethnicities, found ONSD ranges from 2.4 to 7.7 mm in healthy controls (17, 61, 62). In addition, individuals vary in baseline cognitive functions measured by ImPACT scores. For both ONSD and ImPACT scores, changes relative to baseline can offer insight into presence of neurotrauma, especially in revealing more subtle patterns that may be present with sub-concussive hits.

Linear Mixed Effects modeling provides a way to account for within-subject variability across longitudinal repeated measures. In so doing, an LME model normalizes for individual differences in innate physiology and lifestyle that may otherwise impose a confounding influence on oculomotor, ONSD, and ImPACT measurements. Physiologically, subjects' baselines vary, as well as individual susceptibility and resilience to brain trauma (30, 63). Differences in neurochemistry (64), metabolic function, and genetic makeup (44, 65) all contribute to these individual differences. Taken together, there are a host of individual factors that affect susceptibility and recovery following neurotrauma. In the present study, within-subject normalization brought to light that changes observed through the months of participation in contact sports are clear but subtle, underlining the importance of accounting for this individual variability.

### 5.4. Limitation and Future Work

The inability to accurately count the number of sub-concussive impacts as well as their severity remains a challenge. Progress has been made in using helmet-mounted accelerometers (66), but the data from the devices can depend on the fit of the helmet, the location of the hit, and the position of the accelerometers (67, 68). False alarms can be registered on the accelerometers if the helmets are dropped or harshly handled when not being worn. Additionally, individual physiological variability could lead to highly variable outcomes despite the same physical insult. Therefore, the ability to gain a “truth” about the nature and number of sub-concussive hits remains an open problem. In keeping with findings from prior studies (8, 31), the data from the HITS devices used in the present study are reported as averages across the pool of subjects, and minimally used as a way to track an individual's level of head impact exposure.

In the present study, a baseline was needed to accurately model the physiological change. A limitation of the present work is that an initial estimate was always needed for the subjects, which may hinder future diagnostics wherein no prior data is present for that individual. In future work, the models can be extended to predicting completely new data. The challenge remains that the within-subject modeling, enabled using the LME model, was key to making predictions and therefore, the strength of the present work is more in tracking changes within individuals, as opposed to predicting the state of a totally new individual. Solutions to this challenge could be to build on the existing models and collect a single baseline value for each subject. Then, the model predictions could be used in a proactive sense to help make return-to-play decisions.

In the subject pool, all the male subjects were football players and all the female subjects were soccer players. As such, disambiguating the interaction been gender and sport would be impossible. Given the sample size of subject pool, separately analyzing data from the football and soccer players would reduce statistical power. Nevertheless, quantifying the effect of head impact exposure across sports and genders is an important area of ongoing research (37, 69). In future work, a larger study, either standardizing the sport/gender or having a larger sample across sports/genders, will be needed.

## 6. Conclusion

Repetitive sub-concussive head impact exposures have the potential to cause physiological and neurocognitive performance changes. In this study, oculomotor features are used to predict both ONSD and ImPACT scores. Models showed capability for robustly capturing information related to physical (ONSD) and functional (ImPACT) changes. The specific features that were predictive relate to motor control and visual-motor processing. Future work will be needed to quantify the HAE of contact sport athletes while directly relating those data to physiological changes observed through a season of sport.

## Data Availability Statement

The raw data supporting the conclusions of this article will be made available by the authors, without undue reservation.

## Ethics Statement

The studies involving human participants were reviewed and approved by the Purdue Institutional Review Board (IRB) as well as the Committee on the Use of Humans as Experimental Subjects (COUHES), which is the IRB for the Massachusetts Institute of Technology. The patients/participants provided their written informed consent to participate in this study.

## Author Contributions

TV, JL, TS, and TT performed the data collection. SY, TV, JL, and HR analyzed the data. SY and HR wrote the manuscript. JW, TT, and KH provided the feedback on the analysis and manuscript. TQ oversaw all aspects of the program. All authors contributed to the article and approved the submitted version.

## Conflict of Interest

The authors declare that the research was conducted in the absence of any commercial or financial relationships that could be construed as a potential conflict of interest.
