# Supplementary material for: Using Oculomotor Features to Predict Changes in Optic Nerve Sheath Diameter and ImPACT Scores From Contact-Sport Athletes
Source: Front Neurol. 2021 Mar 4;12:584684. doi: 10.3389/fneur.2021.584684 (PMC7969804; doi:10.3389/fneur.2021.584684)
Supplement: Supplementary file 1 [file Data_Sheet_1.pdf]

Using Oculomotor Features to Predict Changes in Optic Nerve Sheath Diameter and ImPACT Scores from Contact-Sport Athletes

Hrishikesh M. Rao [1,*], Sophia Yuditskaya [1], James R. Williamson [1], Trina R. Vian [2], Joseph J. Lacirignola [2], Trey E. Shenk [3], Thomas M. Talavage [4,5], Kristin J. Heaton [6], and Thomas F. Quatieri [1]

[1] *Human Health & Performance Systems Group, Massachusetts Institute of Technology Lincoln Laboratory, Lexington, MA, United States*
[2] *Counter-Weapons of Mass Destruction Systems Group, Massachusetts Institute of Technology Lincoln Laboratory, Lexington, MA, United States*
[3] *Advanced Radio Frequency Techniques & Systems Group, Massachusetts Institute of Technology Lincoln Laboratory, Lexington, MA, United States*
[4] *Department of Biomedical Engineering, Weldon School of Biomedical Engineering, Purdue University, West Lafayette, IN, United States*
[5] *Department of Electrical and Computer Engineering, Purdue University, West Lafayette, IN, United States*
[6] *Military Performance Division, U.S. Army Research Institute of Environmental Medicine, Natick, MA, United States*

*Correspondence:
Hrishikesh M. Rao
hrishikesh.rao@LL.mit.edu

1 SUPPLEMENTARY CONTENT

The hits are thresholded to ≥20g, which provides a reasonable estimate of the presence of a head acceleration event (HAE) (31). In **Supplementary Figure. 1A**, the count of HAEs are limited to just the week preceding the time the ONSD measurement is taken. This slice of data is meant to reflect the immediately sustained head exposure as it relates to the immediate changes in ONSD. In **Supplementary Figure. 1B**, the count of HAEs are reported as the cumulative head exposure leading up to the week preceding the ONSD measurement date. These data are meant to reflect how the cumulative and sustained head impact exposures might contribute to the ONSD measurement. In both cases, there is a positive slope to the regression line, but neither slope is significant. While the HITS data provide reasonable estimates of aggregate head exposure data on the population level, there are known limitations associated with the estimates of the acceleration of individual subjects on shorter time scales (31).

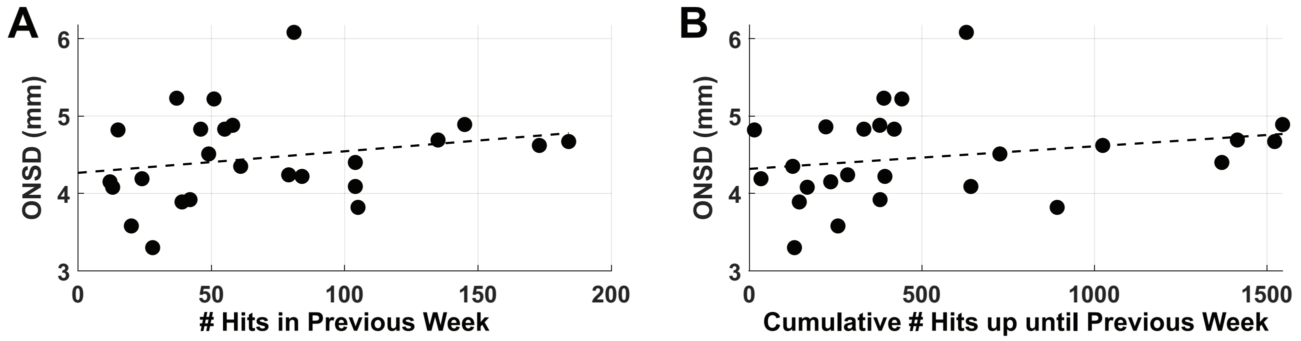

Supplementary Figure 1. Correlations between number of HAEs and ONSD. **(A)** ONSD values were correlated with the number of HAEs sustained by the individual in just the week preceding the measurement of ONSD. The correlation (Corr = 0.231) and the slope of the regression line (Slope = 0.0028) are not statistically significant ($p = 0.26$). **(B)** ONSD values were correlated with the cumulative number of HAEs sustained in the weeks leading up to that ONSD measurement. The correlation (Corr = 0.234) and the slope of the regression line (Slope = 0.0003) are not statistically significant ($p = 0.25$).

Oculomotor Feature Type	Eye-tracking Tasks	Statistics Computed	Description
**Fixation Dispersion**	VG, AS	Median, Stdev	Dispersion of each fixation, measured as the average distance of gaze positions during the fixation from its centroid.
**Inter-Saccade Interval**	SP	Median, Stdev	Median duration between successive saccades during smooth pursuit.
**Intervening Saccade Frequency**	SP	Count	Number of saccades during smooth pursuit.
**Normalized Error of First Saccade**	VG, AS	Median, Stdev	For each target, its normalized Euclidean distance to the first saccade made after it appeared. Normalization divides the computed Euclidean distance error by the difference between the current and immediately preceding target locations.
**Number of Premature Saccades**	VG, AS	Count	Number of premature saccades made during each target.
**Number of Targets with Premature Saccades**	VG,AS	Count	Number of targets for which there was at least one prematurely broken fixation.
**Position Root Mean Squared Error**	VG, AS, SP	Median, Stdev	Root mean squared error of raw eye gaze position relative to concurrent target location.
**Position Root Mean Squared Error Learned**	VG, AS, SP	Median, Stdev	Difference between root mean squared error of raw eye gaze position for first and last targets, normalized by baseline root mean squared error of eye position for first target.
**Reaction Time**	VG, AS, SP	Median, Stdev	Latency of the first saccade to the target after each new target appears.
**Saccade Error**	VG, AS	Median, Stdev	Euclidean distance of each saccade to concurrent target.
**Saccade Q-Ratio**	SP	Median, Stdev	(Peak Velocity x Duration)/Amplitude
**Saccades to Minimum Fixation Distance**	VG, AS	Count	Number of saccades to each fixation from its nearest preceding target.
**Slope of Main Sequence**	VG, AS, SP	Median, Stdev	Slope of the saccade “Main Sequence” (velocity:duration relationship).
**Time to Fixate on Target**	VG, AS	Median, Stdev	Time to reach minimum fixation distance from the nearest preceding target.
**Velocity Gain**	SP	Median, Stdev	The ratio of eye velocity to target velocity.

Supplementary Table 1. Summary of Oculomotor Features. Tasks in which the features are computed are listed as Visually-Guided saccade (VG), Anti-Saccade (AS), and Smooth Pursuit(SP) tasks.
